# The Feasibility of a Web-Based Educational Lifestyle Program for People With Multiple Sclerosis: A Randomized Controlled Trial

**DOI:** 10.3389/fpubh.2022.852214

**Published:** 2022-04-27

**Authors:** William Bevens, Tracey J. Weiland, Kathleen Gray, Sandra L. Neate, Nupur Nag, Steve Simpson-Yap, Jeanette Reece, Maggie Yu, George A. Jelinek

**Affiliations:** ^1^Neuroepidemiology Unit, Melbourne School of Population and Global Health, The University of Melbourne, Carlton, VIC, Australia; ^2^Centre for Digital Transformation of Health, The University of Melbourne, Parkville, VIC, Australia; ^3^Menzies Institute for Medical Research, University of Tasmania, Hobart, TAS, Australia

**Keywords:** ehealth, digital health (ehealth), lifestyle, multiple sclerosis, education

## Abstract

**Background:**

Modifiable lifestyle factors are important to aid people with multiple sclerosis in the self-management of their disease. Current self-management programs are limited by their face-to-face mode of delivery but there is immense potential with the internet to deliver these programs effectively.

**Objective:**

The aims of this study are to assess the feasibility of a digitalized educational lifestyle self-management program for people with MS.

**Methods:**

In this randomized controlled trial, people with MS were randomly allocated to participate in a 6-week tailored web-based educational lifestyle program or 6-week generic standard-care educational course, and were blinded to their allocation. Participants were recruited through multiple sclerosis (MS) Societies in four countries: Australia, New Zealand, Canada, and the United States. The primary outcome was to assess acceptability of the program defined as percentage completion of all modules at 6-weeks post-course commencement. Secondary outcomes included evaluating participant responses to the follow-up survey across three domains: accessibility, learnability, and desirability.

**Results:**

Thirty-five participants from Australia, Canada, New Zealand, and the US completed the baseline survey and were randomized. Four participants were deemed ineligible due to incomplete baseline data; therefore, nine out of 15 and eight out of 16 participants completed 100% of the course in the intervention and standard-care arm courses, respectively.

**Conclusions:**

This study found that this web-based educational lifestyle program is a feasible means of delivering educational content to people with MS *via* the internet according to our a priori targets of >40% of participants in the intervention arm, and >25% in the control arm to completing 100% of the course. It is therefore appropriate to evaluate this intervention further in a large, randomized controlled trial.

**Trial registration:**

This study was prospectively registered with the Australian New Zealand Clinical Trials Registry (ID: ACTRN12621000245897).

## Introduction

Multiple sclerosis (MS) is an autoimmune inflammatory and degenerative disorder of the central nervous system, the clinical outcomes of which are extremely variable (1). Modifiable lifestyle factors are of increasing interest to people with MS with good evidence to support their role in disease management. Factors including diet, omega 3 and vitamin D supplementation, physical activity, and meditation have been associated with improved quality-of-life (QoL) and reduced depression risk in people with MS (2–7). Education is a key step of the process by which people with MS are able to implement this evidence into the management of their condition (8). This requires a structured education program where relevant evidence can be delivered that facilitates adoption of lifestyle changes.

An educational lifestyle program has been delivered in-person to people with MS internationally since 2002 and its recommendations outlined in print (9). Longitudinal follow up has demonstrated that participant adherence to recommended behavior changes for at least 5 years was associated with improved health related QoL and reduced depression risk and fatigue (3, 6, 10, 11). A randomized controlled trial (RCT) of the health outcomes of this educational lifestyle program is an important next step to test the effectiveness of this mode of health intervention. However, the intensive residential delivery mode of this program presents multiple access barriers for people with MS and limits participant numbers, so a new delivery mode for this program is needed to increase participation and enable effectiveness research. Thus, in 2017, the authors began development of the Multiple Sclerosis Online Course (MSOC), the transformation of the in-person educational lifestyle program into a web-based format.

Before health outcomes of the MSOC program can be assessed in an effectiveness study, feasibility testing is required to determine the web-based program's acceptability to users. Acceptability refers to users' perceived value of a technology (12) and mediates how and to what extent they engage with it (13). This is particularly important in self-management interventions where users need to engage with an intervention to acquire knowledge and develop skills to facilitate positive behavior change (8). For online self-management interventions, accessibility remains a key consideration for people with MS (14). Further, the ability to quickly familiarize oneself with the technology (learnability) and the overall enjoyment of that technology (desirability) are important factors in determining the acceptability for users (15). These factors not only influence the likelihood that a user will both commence and complete a program but also whether they are able to derive information necessary for self-management.

Therefore, as preliminary to an RCT of MSOC effectiveness, the aims of this study are to assess the acceptability of the web-based delivery of an educational lifestyle program, and thus its likelihood of attracting substantial participant numbers, and to explore variables such as recruitment and completion of the baseline survey, to inform sample size and recruitment strategy for the effectiveness study.

## Methods

### Ethics

This study was approved by The University of Melbourne Human Research Ethics Committee (ID: 1851781). Written informed consent was obtained from all participants prior to inclusion. This study was prospectively registered with the Australian New Zealand Clinical Trials Registry (ID: ACTRN12621000245897). This study is reported in accordance with the CONSORT-EHEALTH guidelines to ensure sufficient details of the intervention and study design are described (16).

### Trial Design

This study was a parallel group trial design RCT with a 1:1 allocation ratio.

### Participants

Participants were aged 18 years or older; with MS diagnosed by a physician; proficient in English, and residing within Australia, Canada, New Zealand, UK, or USA.

### Recruitment

Participants were recruited through national MS society websites in the USA, New Zealand, and Canada, and within a Facebook patient support group for people with MS in Australia. The national and local MS societies in the UK were unable to participate for this study. Advertisements were active from the 3rd of March 2021 on the USA website, 9th of March on the Australian Facebook group, 24th of March on the New Zealand website, and the 25th of March on the Canadian website.

### Consent

Advertisements contained a link to the study landing page (https://www.msonlinecourse.com.au/) where the plain language statement was presented. After this information, a signup form was presented that required completion of name and email address details, and questions assessing inclusion criteria. Those meeting the inclusion criteria were automatically registered participants as “pending,” and sent a baseline survey to capture demographic, health behavior, and health outcome data. Consent to participate in the study was confirmed on the first page of the baseline survey.

### Outcomes

The primary outcome of this feasibility study was the acceptability of the web-based delivery of an evidence-based educational lifestyle program, determined by number of participants that completed the course. Feasibility was set at >40% of participants in the intervention arm, and >25% in the control arm to complete all the modules. These targets were chosen based on similar online studies in MS (17–19) and likelihood of greater attrition with the standard care arm.

The secondary outcome measure was to assess the mechanism of acceptability *via* the domains, accessibility, learnability and desirability, which were adapted from a comparable feasibility study of a mobile health intervention (15). Accessibility related to font size, ease-of-interaction with the interface, ease-of-navigation; learnability related to ease of learning how to complete expected tasks, and desirability related to enjoyment derived. The tertiary outcome measures were to assess recruitment of participants and percentage completion of the pre-course baseline survey.

### Randomization and Allocation

Upon survey completion, participants' accounts became active, and simple randomization automatically occurred. The randomization sequence was computer-generated and implemented through the course website where researchers had no control over this process. Participants were allocated to one of two study arms: an intervention course (the intervention arm) or an active comparator course (the standard-care control arm). Including an active comparator course in this feasibility study is important to assess its acceptability alongside the intervention. An active comparator is required for medium-to-long term follow-up in a future effectiveness study where a wait-list control may not be feasible or ethical (20). Active comparator control arms have been successfully used in similar online RCTs for people with MS (21, 22). Participants were blinded to allocation but researchers were not. Participants were emailed account details which allowed login, account set up, and access to their allocated course through the website at the URL provided.

Each course was delivered through the same website. Both arms were identical in their format and template designs, the only difference being the content. Each course had seven modules: introduction, diet, physical activity, sunlight and vitamin D, stress reduction, family, and concluding remarks. Each module had a range of different content types: text, video/animation, audio, interactive tasks, and quizzes. Each participant could create a profile that could be viewed by other participants. There was a moderated forum in both intervention and standard-care arms where participants could ask and answer questions.

### Intervention Arm Content

Content in the intervention arm was adapted from the evidence-based lifestyle modification program for people with MS (9); recommendations are summarized below (Table 1).

**Table 1:** Content and recommendations of the intervention and standard care arms.

**Week**	**Modules**	**Intervention arm content and recommendations**	**Standard care arm content and recommendations**
1	Introduction	Introductions of course practitioners and tutorial on course functionalities, format, and timing	Introductions of course practitioners and tutorial on course functionalities, format and timing
	Diet and dietary supplementation	A plant-based wholefood diet plus seafood, with very low saturated fat (<20 g/day) No dairy, meat, palm or coconut oil Omega-3 fatty acid supplements: 20–40 mls of flaxseed oil daily Optional B-group vitamins or B12 supplement No tobacco smoking and avoid passive smoking Moderate alcohol consumption permissible	Eat a balanced diet and follow your national guidelines: Public Health England Eatwell guide presented. Common diets presented: gluten free, Paleolithic diet, McDougal diet, Mediterranean diet. Alcohol consumption should follow national guidelines
2	Vitamin D and sunlight	Sunlight 15 min daily 3–5 times a week as close to all over body exposure as practical Vitamin D3 supplement of at least 5,000 IU daily, adjusted as directed by physician to keep serum levels of vitamin D high, between 150 and 225 nmol/L 25-hydroxyvitamin D (may require up to 10,000 IU daily)	No specific recommendations on sun exposure or supplementation described. Three options presented: wait until more information is available, supplement “blindly” or supplement if blood 25-hydroxyvitamin D levels are low.
	Physical activity	20–30 min, roughly five times per week, preferably outdoors	30 min of moderate intensity aerobic activity and strength training twice per week.
3	Stress reduction	30 min daily meditation	No conclusive link between stress and MS
	Family and prevention	Education on the genetic role in MS as it relates to families	Education on the epidemiology of MS as it relates to families
4	Conclusion	Concluding remarks and closing ceremony	Concluding remarks and closing ceremony
5	Catch-up for those that have not completed the modules		
6	

### Standard-Care Arm Content

Content, including text, video, and images, in the standard-care arm was sourced entirely from publicly available MS society websites, including Multiple Sclerosis Australia, Multiple Sclerosis Research Australia, National MS Society, Multiple Sclerosis Society UK, Multiple Sclerosis Society of Canada. Content mirrored the intervention course with respect to the topics of modules only. Recommendations were general in nature but followed a format of: there is no “best” diet for MS so aim to eat a balanced diet and follow your national guidelines; physical activity is safe and is encouraged for people of all physical abilities twice per week; there is insufficient evidence that vitamin D supplementation has a role in managing MS progression outcomes; meditation may help reduce stress but there is weak evidence to support link between stress and MS; an overview of the genetics role in MS (Supplementary Table 1).

The number of text, video, and image elements differed between the two courses, which resulted in an intervention arm course with more content in the diet and stress reductions modules. Participants in the intervention arm were also sent a link to an electronic version of the written text prior to study commencement. This e-book served as a course guide, which provided further reading and more in-depth discussions around the topics.

### Timing of Delivery

Both arms were conducted simultaneously and in parallel with each other for 6 weeks from the 26th of April to June 7th, 2021. Modules were released sequentially twice per week at 12AM AEST and participants were required to complete each module before advancing to the next. After the final module was released, participants had two further weeks to complete all modules. Participants were sent reminders 1 month before the course commenced if their profile was not complete, the day of each module's release, and were sent a reminder to complete modules that were incomplete at the release of a new module.

### Design and Development

The MSOC was developed by GAJ, TW, SN, and WB, an industry development team (JMAcreative: https://jmacreative.com.au/), and a community advisory group (CAG) of people with MS from Victoria, Australia. The research team was responsible for the content design and development, study design decisions, and final approval of all material.

CAG participants were recruited by advertising for people with MS to contribute to the design of an online lifestyle education program through an Australian Facebook group “People Living with MS.” Discussions with the five members centered around and informed four broad themes: content, format/layout, timing/pacing and engagement. Annotated transcripts from the discussions are available in the Supplementary Files. Key decisions around peer-to-peer forums, study length, and module release frequency, color scheme, and compulsory content completion were decided from these discussions.

The design and development of the course adhered to the principles of the Center for e-Health Research and Disease Management (CeHReS) roadmap (23). This development framework is based upon key principles of human-centered and persuasive technology designs whereby the use of technology should facilitate behavior change. This iterative framework contains five discrete steps that were implemented in our design and development process: contextual inquiry, value specification, design, operationalization, and summative evaluation. This process is described more fully in Supplementary Materials.

### Data Collection

After indicating consent for study participation, participants were requested to complete a baseline survey. Baseline (and subsequently follow-up) data were collected as a long-form survey from the online survey software, Qualtrics (Qualtrics, Provo, USA; Supplementary Figure 1). The survey used validated self-report tools and researcher-devised items where existing tools were not available.

#### Baseline Survey

The baseline survey gathered demographic, health behavior and health outcome data, briefly described. Health outcome data were collected to mirror the baseline survey intended for use in a future RCT of efficacy and determine its rate of completion.

##### Demographics

We collected the following self-reported data from participants: sex; gender identity; country of residence; year of birth; weight; height; marital status; number of children; MS-type: relapsing-remitting MS (RRMS); secondary progressive MS (SPMS); primary progressive MS (PPMS); progressive-relapsing MS (PRMS).

##### Health Outcomes

QoL was measured *via* MSQOL-54 (24); disability by Patient-Determined Disease Steps (PDDS) (25); self-efficacy by the University of Washington Self-Efficacy Scale (26); fatigue by the Fatigue Severity Scale (FSS) (27); depression-risk by the Patient Health Questionnaire 9 (PHQ-9) (28); perceived social support by the Multidimensional Scale of Perceived Social Support (MSPSS) (29); level of visual impairment by the vision component of the validated Performance Scale (PS-V) (30).

##### Modifiable Lifestyle Factors

Diet was assessed using a modified version of the Diet Habits Questionnaire (DHQ) (31) as described previously (32); physical activity by the Godin-Shepherd Leisure-Time Physical Activity Questionnaire (33, 34); sun exposure, vitamin D supplementation, stress reducing activities, alcohol intake, comorbidities, and medication use were assessed using researcher-devised tools reported previously (35).

### Materials for Follow-Up Data

One day after course completion, all participants regardless of completion, were surveyed across three domains: course *accessibility, learnability*, and *desirability*, scored on 3- or 5-point Likert scales. Data are presented in **Figures 2A–C**. Supplementary Figure 2 includes the full survey.

### Analytics Data

Data on recruitment and attrition were derived from within the course functionality, and presented as percentages. Access of the course was measured using Google Analytics, which presented time and date of access, session length, and number of sessions per user.

### Statistical Analysis

As there is no best-practice for sample size calculations in feasibility studies, the proposed sample size was determined with consideration that a minimum of 12 participants per arm is considered adequate to measure acceptability (36) while 30 participants per arm is considered suitable to estimate an effect size (37). The maximum recruitment number of 100 per arm was decided by the research team as greater numbers would impact the social aspects of the course.

Demographics, lifestyle factors, and health outcomes are presented as descriptive statistics (Table 2). Continuous variables were first assessed for normality using the Shapiro-Wilk test. Continuous data are summarized by means and standard deviations for normally-distributed data, and median and interquartile range (IQR) for skewed data. Categorical data are summarized by frequencies with percentages. Baseline measures were tested for statistical significance across arms to validate the randomization process, which included independent samples *t*-test, Chi-square test, and Kruskal–Wallis test, as appropriate. Data were analyzed using Stata, V16 (StataCorp, College Station, USA).

**Table 2:** Characteristics of participants (*n* = 31).

**Characteristic**	**Intervention course**	**Standard-care course**	***p***
	**(*n* = 15)**	**(*n* = 16)**	
Age (years)	52 (11.5; 32–75)	53 (10.4; 35–67)	0.90
mean (SD; range)			
Sex, *n*			
Female	11 (73.3%)	13 (81.3%)	0.60
Male	4 (26.7%)	3 (18.7%)	
Gender, *n*			
Female	11 (73.3%)	13 (81.3%)	0.60
Male	4 (26.7%)	3 (18.7%)	
Country, *n*			
Australia	4 (27%)	8 (50%)	0.83
Canada	1 (7%)	2 (12%)	
New Zealand	6 (40%)	6 (38%)	
USA	4 (26%)	0 (0%)	
MS type, *n*			
RRMS	11 (73.3%)	11 (69%)	0.82
SPMS	1 (6.7%)	2 (12%)	
PPMS	2 (13.3%)	3 (19%)	
Missing	1 (6.7%)	0 (0%)	
Time since	12 (10.3)	14 (12.1)	0.60
diagnosis (years)			
median (IQR)			
Missing, *n*	1 (6.7%)	0 (0.0%)	
PDDS, *n*			
Normal/mild	4 (26.6%)	8 (50.0%)	0.22
Moderate	7 (46.8%)	3 (18.7%)	
Severe	4 (26.6%)	5 (31.3%)	

*p, p-value; SD, standard deviation; n, number; IQR, median and interquartile range; PDDS, patient determined disease steps; Statistical significance tests performed were independent samples t-test for normally-distributed means, Kruskal–Wallis for skewed data, and Chi-square for associations between categorical variables and arms*.

## Results

### Participant Cohort

**Figure 1:**
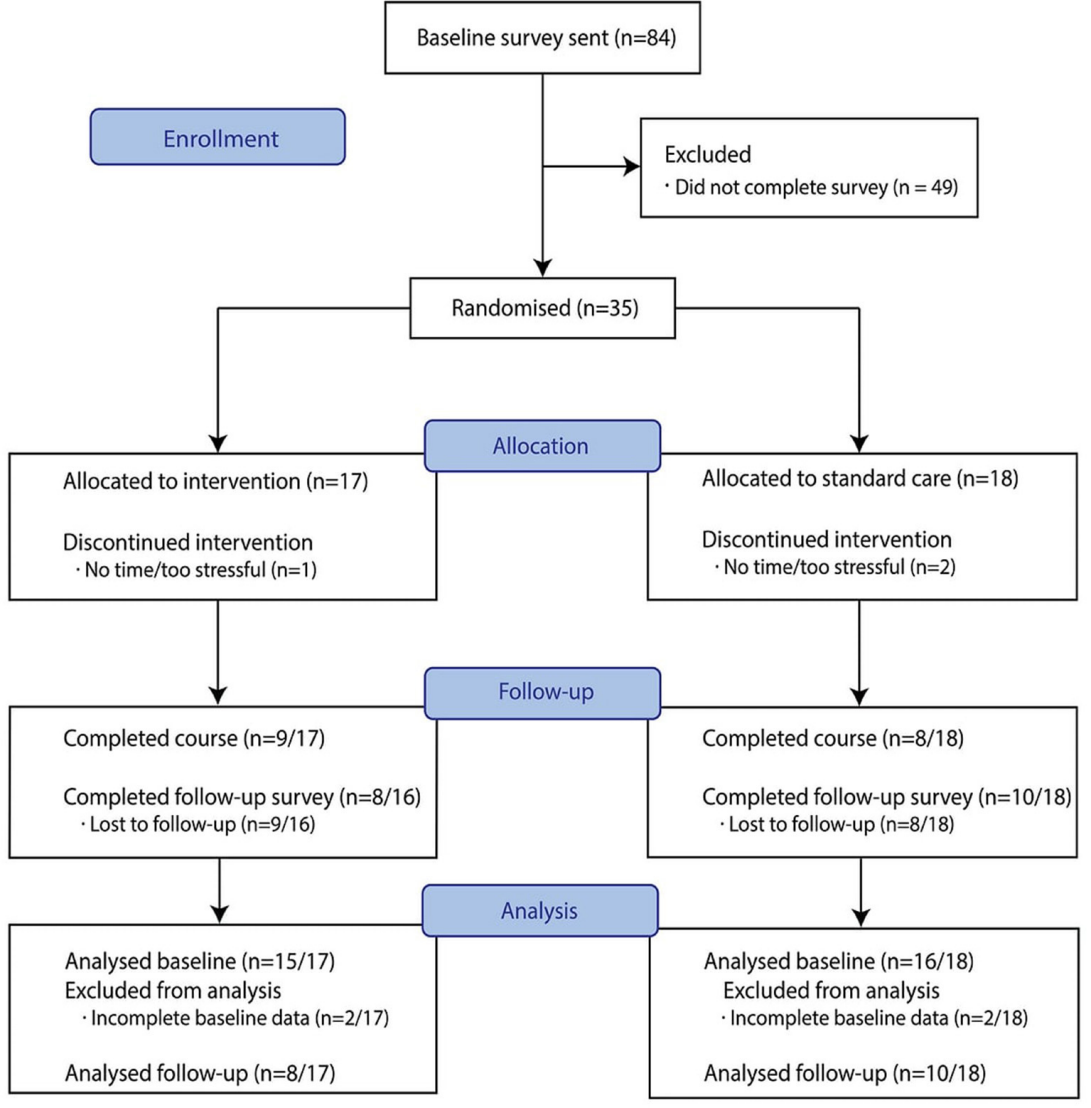
Consort flow diagram.

A total of 84 people with MS were sent a baseline questionnaire (Figure 1) and 35 participants were randomly allocated: 17 to the intervention arm and 18 to the control arm. Two participants from each arm had incomplete survey data upon post-study period analysis, rendering them ineligible for analysis. Therefore, 31 are considered to have completed the baseline survey. None of these four ineligible participants commenced the course and were excluded from analysis. The mean time for survey completion was 69 min (SD = 13·7; 25–387).

The participant cohort was made up of ~3:1 female to male in the intervention group and 4:1 in the standard-care group. Most participants resided in Australia and New Zealand (77%). Most participants in the intervention [11 (79%)] and standard-care [11 (69%)] arms reported having RRMS.

### Course Completion

The course was fully completed by nine and eight participants in the intervention and standard-care arms, respectively (Table 3). No participants commented in the forums in either arm. Three participants in the intervention arm and one in the standard-care arm did not complete any of the course.

**Table 3:** Percentage of course completed.

	**Intervention (*n* = 15)**	**Standard-care (*n* = 16)**
Completion, *n(%)*		
7/7 modules	9 (59%)	8 (50%)
6/7 modules	0 (0%)	1 (7%)
5/7 modules	1 (7%)	1 (7%)
4/7 modules	0 (0%)	1 (7%)
3/7 modules	1 (7%)	1 (7%)
2/7 modules	0 (0%)	3 (20%)
1/7 modules	1 (7%)	0 (0%)
Never commenced	3 (20%)	1 (7%)

### Quantitative Follow-Up

Eighteen of 31 participants (58%) returned follow-up surveys: eight from the intervention arm (53%) and 10 from the standard-care arm (63%). Of those who completed the follow-up survey, 14 completed 100% of the course, three partially completed the course, and one completed only the first module.

**Figure 2:**
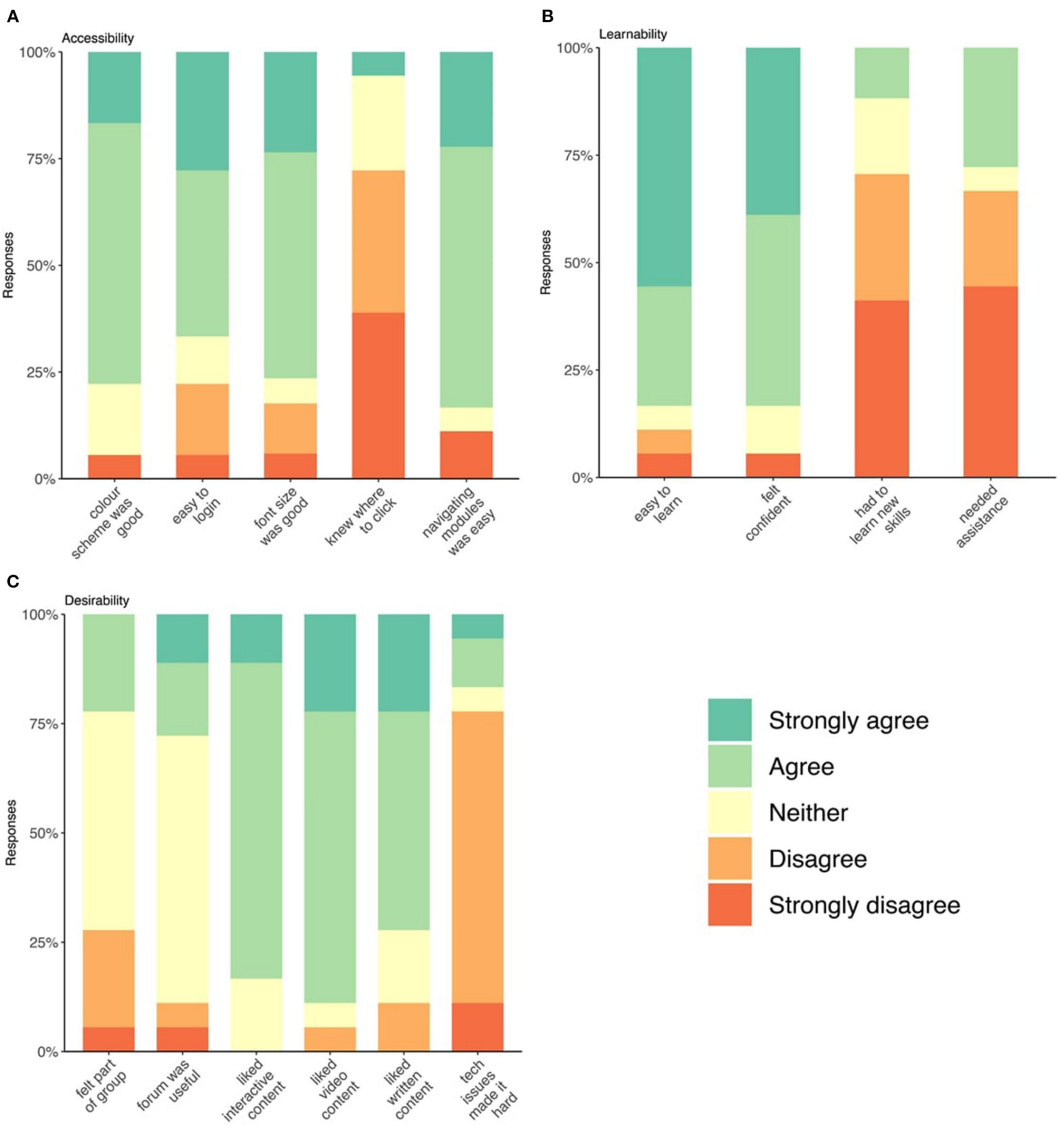
**(A–C)** Stacked bar charts of respondent's answers to follow-up Likert survey on accessibility, learnability, and desirability of the course.

Under the *accessibility* domain, 12 (66%) respondents found it easy to login to the MSOC while four respondents described difficulties (Figure 2A). Most participants found navigating the modules easy, and 14 (78%) and 13 (72%) found the color scheme and font-size appropriate, respectively. However, 5 (28%) respondents reported sometimes or always having difficulty finding where to “click.” Under the *learnability* domain (Figure 2B), 15 (83%) of respondents found the course easy to learn but 5 (28%) reported having required assistance from the research team during the course. Under the *desirability* domain, all content types (video, text, interactive) were well-received across both arms. Half the respondents neither agreed nor disagreed that they felt part of a group during the course (Figure 2C).

No participants desired the course to be shorter than 6 weeks (Supplementary Table 2), and the majority 13 (72%) thought the course was of the right length, while five (28%) desired a longer course. Similarly, 15 (83%) thought the release of two modules per week was appropriate, while two (11%) would be happy with a greater frequency and only one respondent preferred lower frequency.

### Analytics

Course website access peaked on the day of module release (Supplementary Figure 3) and most participants completed modules within 2 days of release. The average session time for participants in the was 17 min and 22 s and 13 min for intervention and standard-care arms, respectively, with the time spent on each page averaging just over 1 min for both. The course was accessed *via* desktop primarily but also laptop computer, smart phone and tablet devices, with sessions on tablets lasting far longer when compared with desktop and mobile (Supplementary Table 1).

## Discussion

The internet provides opportunities for people with MS to learn how to manage their disease without the geographical or temporal limitations of location-based learning resources and programs. Further, people with MS access online information and use the internet for healthcare reasons at high rates, suggesting that online supports for self-management are valued by this population (38–40). Digital health interventions have shown efficacy in assisting people with MS in making lifestyle changes likely to benefit overall health (17–19, 41–43). The present study builds on existing work on digital health for people with MS and provides additional insight into factors that affect user engagement and participation with DHIs.

This study evaluated the feasibility of a web-based educational lifestyle program in a cohort of people with MS. Nine (60%) participants in the intervention arm and eight (50%) of participants in the standard care arm completed 100% of the course, which exceeded the feasibility thresholds of >40% and >25% completion in the intervention arm and standard-care arms, respectively. This indicates an adequate level of acceptability of the mode of learning for participants. In general, participants from both arms found the content suitable, and the course accessible and navigable, indicating good acceptability, our secondary outcome. The tertiary outcomes of recruitment and baseline survey completion described a completion rate of 31 people out of 84 (37%).

Our baseline survey completion rate was 37%, while previous online studies have described higher rates with ranges of 39.3–80.5% (18, 19, 44–46); these studies contained fewer outcome measures and were therefore likely shorter and completed more quickly. Interestingly, 35 participants in this study commenced the baseline survey but did not complete it. Data for the reasons for non-completion are not available but future research should seek to understand this phenomenon as this has important impacts on sample size calculations. As baseline survey completion appeared to be a significant barrier to participation, it is important to ensure the baseline survey is shorter but remains adequate to assess outcomes for the future effectiveness study. To our knowledge, there is no gold-standard for baseline survey length, and requirements may vary on a case-by-case basis, as suggested by the large standard deviation in survey completion time described here. Considering our study and others described here (18, 19, 44–46), a baseline survey with three to four outcomes measures should not constitute a barrier to participation. Completion of the baseline survey was hypothesized to act as “buy-in” and encourage participants to complete the course; however, four participants did not complete any of the modules despite completing the baseline survey. It is possible the length of the baseline survey acts as a barrier to entry for this study and novel means of delivering a long-form health outcome survey should be considered to increase accessibility. Analysis did not describe any differences in characteristics between those that did not start the course and those that started or completed the course.

An important indicator of feasibility is course completion. Similar online studies for people with MS have described 79 and 45% completion of intervention and standard-care arms (19), 68 and 45%, respectively, reached individualized session number targets as prescribed (47), and in another study, sessions completion was 87% across all three arms (46). These completion figures are generally higher than those seen in this study and the reasons for these variations are likely complex. One possible contributor to lower rate of completion in this study is the absence of interaction between clinicians/researchers and participants. Compared with other studies where instructors deliver course content in real-time (46), or staff made “check-in calls” (47) or there was communication between psychologists and participants (19), in our study participants only interacted with a course facilitator for technical assistance. Less interaction may influence drop-out numbers (48) and, therefore, incorporating a system of communication may assist in ensuring completion. However, partial completions may also be of importance as some participants in our study, despite having not completed the course, reported making lifestyle changes aligned with the modules they did complete. It is possible these participants were selective about the information they sought.

Quantitative follow-up was completed by 58% of participants, eight from the intervention arm (53%) and 10 from the standard-care (63%), all but one of which completed all modules of the course. Comparable studies described a follow-up survey completion of 72% (46), 86% (22), 80% (18), indicating follow-up was lower than average in our study, which is likely a results of lower completion of the course itself. Responses regarding accessibility indicated that this intervention is easy-to-use overall; however, almost 75% of respondents indicated that knowing where to click was sometimes difficult. This may be a significant limitation to accessibility as the ability to interact with technology is an important mediator of the overall acceptability of that technology (12). Increasing accessibility is a critical endpoint for researchers designing and developing web-based programs for people with MS. Under the learnability domain, many but not all participants found it easy to learn how to navigate the course and felt confident doing so. Confidence is a critical component of self-management and facilitates a person's ability to engage in health behavior change (49). It is possible that participants' confidence in navigating the web-based course may foster their confidence in changing lifestyle behaviors.

Under the desirability domain, content across video, text, and interactivity was well-liked across both arms. While ~3 quarters of respondents reported that technical issues did not make completing the course hard, there remains a quarter of participant for whom this did impact their ability to complete the course. Most respondents felt “neutral” about the forums as they were not utilized in either arm. The importance of peer support in online self-management interventions has been previously demonstrated (50, 51) and is considered integral to the success of this self-management program also. It was hypothesized that peer support would translate into participants feeling part of a community, however the lack of forum usage had the effect of most respondents feeling either neutral or that they were not part of a group it. This lack of peer support within the course may have impacted its acceptability to participants and, therefore, their engagement with the program. To our knowledge, no other comparable study has implemented and evaluated a forum in a population of people with MS; however, it is possible that instructor-led conversations may promote engagement in the forum as observed in higher education (52). Similarly, in a meta-analysis of online mindfulness-based interventions, significantly greater adherence and effect sizes were seen for stress and mindfulness with therapist guidance in the interventions (53).

Participants found both the length of the course and the weekly release schedule of modules acceptable. Many similar studies report intervention lengths between eight and 12 weeks (19, 45–47). However, 4 weeks of content delivery with 2 weeks of catch-up appears to have been desirable in this context. Aside from the time-gated module release, participants were largely in control of how they proceeded through the intervention. Many respondents described having made lifestyle changes due to the course. While it is beyond the scope of this feasibility study to determine degree of behavior change and health outcomes, these findings provide some evidence that both arms resulted in some initial behaviors changes. While numbers were small, the standard-care arm had a larger proportion of respondents making no change suggesting that it may be a suitable comparator for the intervention. Two respondents in the standard-care arm reported making changes that were not due to course material, an important consideration when determining power calculations for future effectiveness studies.

Analytics data documented user engagement and participants in both arms completed modules soon after release. However, there were participants who accessed and completed modules during the final 2-week period after all modules had been released, indicating that a period of “catch-up” appears useful or participants were interested in revising the content. The amount of time standard care arm participants spent on the website per module was 25% less than time spent by intervention arm participants. This difference may introduce variation in outcome measures not accounted for by course content alone. While most participants accessed the course through a desktop device, other participants accessed the course through a tablet or smartphone. The course was not optimized to function on tablet or phone devices and alternate device use should be considered when designing and developing future web-based courses.

### Limitations

A limitation to this study is the lower-than-expected rate of recruitment of participants. We were unable to utilize the MS societies in Australia and the UK as originally planned with multiple factors including the COVID-19 pandemic limiting the ability of some societies to assist with recruitment. Limited follow-up survey completion may have created participant bias with participants who completed the follow-up questionnaire potentially more likely to respond positively and/or enact lifestyle changes. It is possible that conducting this study during the COVID-19 pandemic, which represented a persistent period of crisis, may have impacted uptake of and response to this intervention. A recent study however, suggests that this is not the case and that results from online studies during this period are in fact generalizable to pre-pandemic periods (54).

The strengths of this study include that the population is representative of the general MS population in that they were young to middle aged, majority female, and primarily of RRMS phenotype. The design and development process of this digital health intervention were based upon a well-established development framework. Further, this project was developed in collaboration with CAG, experienced clinicians and researchers in the MS field, and a development team with a wealth of experience in developing accessible online resources adding expertise and lived experience to the study. The design of a standard-care arm is unique, and the successful feasibility of a control course described in this study allows for a future effectiveness study to measure differences in health outcomes due to the difference in course content as opposed to undertaking an online course itself.

## Conclusion

This study evaluated the feasibility of moving to a fully web-based learning program from a residential intensive evidence-based lifestyle program for people with MS. This finding is important as it describes that the delivery of modular, educational content over 6 weeks is feasible, and other self-management programs with a similar format can be successfully delivered in a similar method. In this RCT to test feasibility, both intervention and standard-care arms showed satisfactory completion rates and the follow-up quantitative survey described general satisfaction with the content, length and format of the courses. Therefore, this study demonstrated the feasibility of this digital health intervention, opening the way to an RCT of effectiveness of the program. Importantly, this study identified limitations with peer-to-peer engagement in the web-based course that need to be addressed in a larger RCT examining the effectiveness of a web-based educational program for people with MS. Beyond an effectiveness study, this study provides further understanding of how people with MS engage with web-ased self-management programs and barriers that they may face accessing these programs.

## Data Availability Statement

The raw data supporting the conclusions of this article will be made available by the authors, without undue reservation.

## Ethics Statement

The studies involving human participants were reviewed and approved by the University of Melbourne Human Research Ethics Committee. The patients/participants provided their written informed consent to participate in this study.

## Author Contributions

WB, TW, GJ, and SN were responsible for conceptualization of the web-based course. WB, TW, KG, SN, and SS-Y were responsible for review before study. WB, TW, GJ, SN, and KG were responsible for overall design considerations of the web-based course. WB, GJ, and SN were responsible for course content development such as videos, text and figures. WB, TW, GJ, SN, KG, NN, and SS-Y were responsible for feasibility study design. WB was responsible for project administration and investigation while SN and KG were responsible for supervision during the feasibility study period. WB and SN were responsible for writing of the original draft. WB, JR, and MY were responsible for data visualization during manuscript development. WB and MY have both seen and verified the underlying/raw data. All authors contributed equally to critical revisions of the article and accept final responsibility for the decision to submit for publication.

## Funding

The Neuroepidemiology Unit receives philanthropic funding from Mr. Wal Pisciotta and other donors, which funded William Bevens' Ph.D. studentship. The Overcoming Multiple Sclerosis Charity provided financial support for the technological development of the course *via* JMACreative and funding for the Article Processing Fee. Funders played no role in course development, design or interpretation of results.

## Conflict of Interest

GJ and SN have previously received remuneration for facilitating lifestyle modification workshops. GJ receives royalties from his book “Overcoming multiple sclerosis: the 7-step recovery program.” The remaining authors declare that the research was conducted in the absence of any commercial or financial relationships that could be construed as a potential conflict of interest.

## Publisher's Note

All claims expressed in this article are solely those of the authors and do not necessarily represent those of their affiliated organizations, or those of the publisher, the editors and the reviewers. Any product that may be evaluated in this article, or claim that may be made by its manufacturer, is not guaranteed or endorsed by the publisher.

## Supplementary Material

Supplementary Figure 1 Baseline survey (pre-study period).

Supplementary Figure 2 Follow-up survey (post-study period).

Supplementary Figure 3 Time series plot of users accessing the courses in both intervention and standard-care.

Supplementary Table 1 Devices used to access the courses.

Supplementary Table 2 Respondent answers to follow-up survey on length of course and frequency of module release.
