# Supplementary figures and images for: The Feasibility of a Web-Based Educational Lifestyle Program for People With Multiple Sclerosis: A Randomized Controlled Trial
Source: Front Public Health. 2022 Apr 27;10:852214. doi: 10.3389/fpubh.2022.852214 (PMC9092338; doi:10.3389/fpubh.2022.852214)

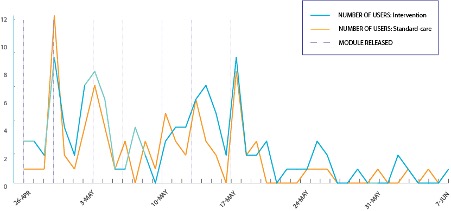

Supplement: Supplementary Figure 1 — Baseline survey (pre-study period). [file Image_1.JPEG]
