# Supplementary material for: The Feasibility of a Web-Based Educational Lifestyle Program for People With Multiple Sclerosis: A Randomized Controlled Trial
Source: Front Public Health. 2022 Apr 27;10:852214. doi: 10.3389/fpubh.2022.852214 (PMC9092338; doi:10.3389/fpubh.2022.852214)
Supplement: Supplementary Figure 2 — Follow-up survey (post-study period). [file Table_1.DOCX]

Outcome Survey

Start of Block: Consent

Q152
I have read and understand the participant information. I freely agree to participate in this project according to the conditions described. I am 18 years of age or older.

- Yes (1)
- No (2)

End of Block: Consent

Start of Block: Demographics

| Page Break |  |
| --- | --- |

Q276 Click to write the question text

- First name (1) ________________________________________________
- Last name (2) ________________________________________________
- Primary email address (3) ________________________________________________
- Primary phone number (please include country and area cod) (5) ________________________________________________

| Page Break |  |
| --- | --- |

Q126 What is your sex?

- Male (1)
- Female (2)
- Indeterminate/Intersex/Unspecified (3)

Q127 What gender do you identify with?

- Male (1)
- Female (2)
- Non binary (4)
- Intersex (5)
- Other (please specify) (6) ________________________________________________

| Page Break |  |
| --- | --- |

Q130 In which country do you reside?

▼ Please select below... (1) ... Other (195)

| 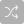 |
| --- |

Q132 What year were you born?

▼ 1920 (1) ... 2000 (81)

| Page Break |  |
| --- | --- |

Display This Question:

If Q130 != United States

Q149 What is your weight?

- Kilograms (4) ________________________________________________
- Pounds (5) ________________________________________________

Display This Question:

If Q130 != United States

Q277 What is your height?

- Centimeters (4) ________________________________________________
- Inches (5) ________________________________________________

| Page Break |  |
| --- | --- |

| 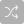 |
| --- |

Q138 What is your current marital status?

- Single, never married (1)
- Married without children (2)
- Married with children (3)
- Divorced (4)
- Seperated (5)
- Widowed (6)
- Living w/ partner (7)

| 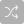 |
| --- |

Q140 How many children do you have (including step-children)?

▼ 0 (1) ... 12+ (13)

| Page Break |  |
| --- | --- |

Q209 What type of MS were you diagnosed with?

- Relapse Remitting Multiple Sclerosis (RRMS) (1)
- Primary Progressive Multiple Sclerosis (PPMS) (2)
- Secondary Progressive Multiple Sclerosis (SPMS) (3)
- Progressive Relapsing Multiple Sclerosis (PRMS) (4)

| 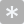 |
| --- |

Q278 What year were you formally diagnosed with MS?

________________________________________________________________

Q115 Have you ever read the book "Overcoming Multiple Sclerosis: an evidence-based guide to recovery" by Professor George Jelinek?

- Yes (1)
- No (2)

End of Block: Demographics

Start of Block: PDDS

Q36
Please read the choices listed below and choose the one that best describes your own situation.

 This scale focuses mainly on how well you walk.

 You might not find a description that reflects your condition exactly, but please mark the one category that describes your situation the closest.

- **Normal**: I may have some mild symptoms, mostly sensory due to MS but they do not limit my activity. If I do have an attack, I return to normal when the attack has passed. (1)
- **Mild Disability:** I have some noticeable symptoms from my MS but they are minor and have only a small effect on my lifestyle. (2)
- **Moderate Disability:** I don't have any limitations in my walking ability. However, I do have significant problems due to MS that limit daily activities in other ways. (3)
- **Gait Disability:** MS does interfere with my activities, especially my walking. I can work a full day, but athletic or physically demanding activities are more difficult than they used to be. I usually don't need a cane or other assistance to walk, but I might need some assistance during an attack. (4)
- **Early Cane:** I use a cane or a single crutch or some other form of support (such as touching a wall or leaning on someone's arm) for walking all the time or part of the time, especially when walking outside. I think I can walk 25 feet in 20 seconds without a cane or crutch. I always need some assistance (cane or crutch) if I want to walk as far as 3 blocks. (5)
- **Late Cane:** To be able to walk 25 feet, I have to have a cane, crutch or someone to holdonto. I can get around the house or other buildings by holding onto furniture or touching the walls for support. I may use a scooter or wheelchair if I want to go greater distances. (6)
- **Bilateral Support:** To be able to walk as far as 25 feet I must have 2 canes or crutches or a walker. I may use a scooter or wheelchair for longer distances. (7)
- **Wheelchair/Scooter:** My main form of mobility is a wheelchair. I may be able to stand and/or take one or two steps, but I can't walk 25 feet, even with crutches or a walker. (8)
- **Bedridden:** Unable to sit in a wheelchair for more than one hour. (9)

End of Block: PDDS

Start of Block: Participant Health Questionnaire

Q208
Over the last 2 weeks, how often have you been bothered by any of the following problems?

Q1 Little interest or pleasure in doing things?

- Not at all (1)
- Several days (2)
- More than half the days (3)
- Nearly every day (4)

Q2 Feeling down, depressed, or hopeless?

- Not at all (1)
- Several days (2)
- More than half the days (3)
- Nearly every day (4)

Q3 Trouble falling or staying asleep, or sleeping too much?

- Not at all (1)
- Several days (2)
- More than half the days (3)
- Nearly every day (4)

| Page Break |  |
| --- | --- |

Q4 Feeling tired or having little energy?

- Not at all (1)
- Several days (2)
- More than half the days (3)
- Nearly every day (4)

Q5 Poor appetite or overeating?

- Not at all (1)
- Several days (2)
- More than half the days (3)
- Nearly every day (4)

Q6 Feeling bad about yourself - or that you are a failure or have let yourself or your family down?

- Not at all (1)
- Several days (2)
- More than half the days (3)
- Nearly every day (4)

| Page Break |  |
| --- | --- |

Q7 Trouble concentrating on things, such as reading the newspaper or watching television?

- Not at all (1)
- Several days (2)
- More than half the days (3)
- Nearly every day (4)

Q8 Moving or speaking so slowly that other people could have noticed?
Or the opposite - being so fidgety or restless that you have been moving around a lot more than usual?

- Not at all (1)
- Several days (2)
- More than half the days (3)
- Nearly every day (4)

Q9 Thoughts that you would be better off dead, or of hurting yourself in some way?

- Not at all (1)
- Several days (2)
- More than half the days (3)
- Nearly every day (4)

Q212
If you or someone you know is feeling suicidal, experiencing a crisis or are in need of immediate help please contact one of your local 24/7 services or some of the local services listed here:
  
Australia: Lifeline on 131 114
 Canada: Toronto Distress Centre 416-408-HELP (4357)
 New Zealand: Lifeline 0800 543 354 (0800 LIFELINE)
 United Kingdom: Samaritans 116 123
 United States: Suicide Prevention and Crisis Hotline 1800-273-8255

End of Block: Participant Health Questionnaire

Start of Block: Fatigue Severity Scale

Q37
Please slide the number between 1 and 7 to where you feel best fits the following statements. This refers to your usual way of life within the last week.

 1 indicates “strongly disagree” and 7 indicates “strongly agree.”

|  | Strongly Disagree | Strongly Agree |
| --- | --- | --- |

|  | 1 | 2 | 3 | 4 | 5 | 6 | 7 |
| --- | --- | --- | --- | --- | --- | --- | --- |

| My motivation is lower when I am fatigued () | 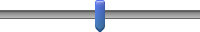 |
| --- | --- |
| Exercise brings on my fatigue. () | 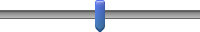 |
| I am easily fatigued () | 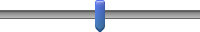 |
| Fatigue interferes with my physical functioning () | 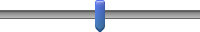 |
| Fatigue causes frequent problems for me () | 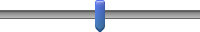 |
| My fatigue prevents sustained physical functioning. () | 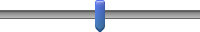 |
| Fatigue interferes with carrying out certain duties and responsibilities. () | 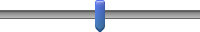 |
| Fatigue is among my most disabling symptoms. () | 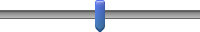 |
| Fatigue interferes with my work, family, or social life. () | 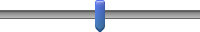 |

End of Block: Fatigue Severity Scale

Start of Block: Comorbidities

Q32 Please select whether or not you have the following:

|  | Do you have the problem? If yes --> | | Do you receive treatment for it? If yes --> | | Does it limit your activites? | |
| --- | --- | --- | --- | --- | --- | --- |
|  | No (1) | Yes (2) | No (1) | Yes (2) | No (1) | Yes (2) |
| Heart Disease (1) |  |  |  |  |  |  |
| High blood pressure (2) |  |  |  |  |  |  |
| Lung disease (3) |  |  |  |  |  |  |
| Diabetes (4) |  |  |  |  |  |  |
| Ulcer or stomach disease (5) |  |  |  |  |  |  |
| Kidney Disease (6) |  |  |  |  |  |  |
| Liver Disease (7) |  |  |  |  |  |  |
| Anemia or other blood disease (8) |  |  |  |  |  |  |
| Cancer (9) |  |  |  |  |  |  |
| Depression (10) |  |  |  |  |  |  |
| Osteoarthritis, degenerative arthritis (11) |  |  |  |  |  |  |
| Back Pain (12) |  |  |  |  |  |  |
| Rheumatoid Arthritis (13) |  |  |  |  |  |  |
| Other medical problems (14) |  |  |  |  |  |  |

End of Block: Comorbidities

Start of Block: Self-efficacy; University of Washington

Q35 How confident are you that:

|  |  | | | | |
| --- | --- | --- | --- | --- | --- |
|  | Not at all (1) | A little (2) | Quite a bit (3) | A lot (4) | Completely (5) |
| You can keep the fatigue caused by your health condition or disability from interfering with the things you want to do? (1) |  |  |  |  |  |
| You can keep the physical discomfort related to your health condition or disability from interfering with the things you want to do?   (2) |  |  |  |  |  |
| You can keep the pain related to your health condition or disability from interfering with the things you want to do? (3) |  |  |  |  |  |
| You can keep the emotional distress caused by your health condition or disability from interfering with the things you want to do? (4) |  |  |  |  |  |
| You can keep any other symptoms or health problems you have from interfering with the things you want to do?   (5) |  |  |  |  |  |
| You can do things other than just taking medication to reduce how much your health condition or disability affects your everyday life?   (6) |  |  |  |  |  |
| You can keep your health condition or disability from interfering with managing your affairs? (7) |  |  |  |  |  |
| You can keep your health condition or disability from interfering with family relationships? (8) |  |  |  |  |  |
| You can keep your health condition or disability from interfering with close friendships? (9) |  |  |  |  |  |
| You can keep your health condition or disability from interfering with your ability to deal with unexpected events? (10) |  |  |  |  |  |
| You can keep your health condition or disability from interfering with your ability to interact socially? (11) |  |  |  |  |  |
| You can keep your health condition or disability from being the center of your life? (12) |  |  |  |  |  |
| You can keep your health condition or disability from interfering with having a fulfilling life? (13) |  |  |  |  |  |
| You can, using all the resources available to you, minimize the occurrence of complications related to your health condition or disability (such as bladder accidents or falls)? (14) |  |  |  |  |  |
| You can bounce back from frustration, discouragement or disappointment that your health condition or disability may cause you? (20) |  |  |  |  |  |
| You can, using all the resources available to you, successfully manage your medication needs? (21) |  |  |  |  |  |
| You can figure out effective solutions to issues that come up related to your health condition or disability? (22) |  |  |  |  |  |
| You can keep your health condition or disability from interfering with having an emotionally intimate relationship with a spouse or partner? (Leave blank if NA) (23) |  |  |  |  |  |
| You can keep your health condition or disability from interfering with having a satisfying sexual relationship? (Leave blank if NA) (19) |  |  |  |  |  |

End of Block: Self-efficacy; University of Washington

Start of Block: Perceived Social Support

Q37
We are interested in how you feel a bout the following statements. Read each statement carefully, and indicate how you
feel about each statement. Move the slider with the mouse to the corresponding numbers:
  
1 - if you Very Strongly Disagree
2 - if you Strongly Disagree
3 - if you Mildly Disagree
4 - if you are Neutral
5 - if you Mildly Agree
6 - if you Strongly Agree
 7 - if you Very Strongly Agree

|  | Very Strongly Disagree |  | Very Strongly Agree |
| --- | --- | --- | --- |

|  | 1 | 2 | 3 | 4 | 5 | 6 | 7 |
| --- | --- | --- | --- | --- | --- | --- | --- |

| There is a special person who is around when I am in need. () | 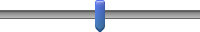 |
| --- | --- |
| There is a special person with whom I can share my joys and sorrows. () | 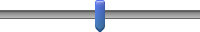 |
| My family really tries to help me. () | 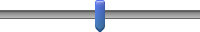 |
| I get the emotional help and support I need from my family. () | 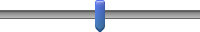 |
| I have a special person who is a real source of comfort to me. () | 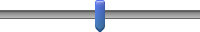 |
| My friends really try to help me. () | 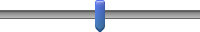 |
| I can count on my friends when things go wrong. () | 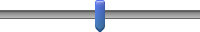 |
| I can talk about my problems with my family. () | 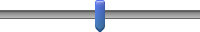 |
| I have friends with whom I can share my joys and sorrows. () | 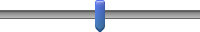 |
| There is a special person in my life who cares about my feelings. () | 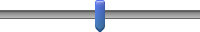 |
| My family is willing to help me make decisions. () | 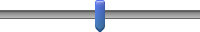 |
| I can talk about my problems with my friends. () | 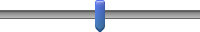 |

End of Block: Perceived Social Support

Start of Block: Dietary Habits Questionnaire

Q57 How many days a week do you eat a high fibre breakfast cereal? (e.g. rolled oats, Weet-bix TM, Allbran TM, untoasted muesli)

- Never, or hardly ever (1)
- Less than once a week (2)
- Once of twice a week (3)
- Three to five times a week (4)
- Six or more times a week (5)

Q58 How often do you eat or use wholemeal or wholegrain bread in preference to white bread?

- Never (1)
- Rarely (2)
- Occasionally (3)
- Usually (4)
- Always (5)

Q59 How often do you eat cereal e.g. pasta, rice, noodles, cous cous, as part of your main meal?

- Never (1)
- Rarely (2)
- Once or twice per week (3)
- Three to four times per week (4)
- Five or more times per week (5)

| Page Break |  |
| --- | --- |

Q60 How many serves of vegetables would you eat in a typical day?

- Five or more serves (1)
- Three to four serves (2)
- One or two serves (3)
- Less than one serve (4)
- None (5)

Q61 How many times a week do you eat two or more pieces of fruit a day?

- Six or more times per week (1)
- Three to five times per week (2)
- Once or twice per week (3)
- Less than once per week (4)
- Never, or hardly ever (5)

Q62 How many days a week do you eat legumes? (e.g. chickpeas, baked beans, three bean mix, lentils, split peas, dried beans etc)

- Six or more times per week (1)
- Three to five times per week (2)
- Once or twice per week (3)
- Less than once per week (4)
- Never, or hardly ever (5)

| Page Break |  |
| --- | --- |

Q63 How often do you include raw nuts or seeds such as pepitas, sunflower seeds or linseeds?

- Five or more times per week (1)
- Three to four times per week (2)
- Once or twice per week (3)
- Rarely (4)
- Never (5)

Q64 When having milk, yoghurt or cheese (from animal sources), how often do you eat or use reduced-fat or low fat products in preference to regular products?

- Always (1)
- Usually (2)
- Occasionally (3)
- Rarely (4)
- Never (5)
- I don't consume dairy (6)

Q65 How many days a week do you eat fish?

- Three or more days per week (1)
- Two days per week (2)
- Once per week (3)
- Less than once per week (4)
- Never, or hardly ever (5)

| Page Break |  |
| --- | --- |

Q66 If you use a spread on bread or cracker biscuits, which type of spread would you usually use?

- Avocado (2)
- Margarine (6)
- Cream cheese (3)
- Butter (4)
- I don't use spreads (5)

Q67 How many days a week do you eat processed meats (e.g. bacon, sausages, salami, ham, frankfurts, or pate)?

- I don't eat meat (1)
- Never, or hardly ever (2)
- Less than once per week (3)
- Once per week (4)
- Two or three times per week (5)
- Four or more times per week (6)

Q68 What type of salad dressing do you normally use?

- Mono/polyunsaturated oil base (1)
- Reduced fat commercial dressing (2)
- Full fat commercial dressing (3)
- I don't use dressing on salads (4)

| Page Break |  |
| --- | --- |

Q69 What type of cooked sauces do you normally use? (You may select more than one.)

- Vegetable/tomato-based sauces (1)
- Reduced fat milk based (2)
- Gravy from commercial powder (3)
- Gravy from pan dripping (4)
- Cream or full cream milk based (5)
- Sauces with coconut milk (6)
- I don't use cooked sauces (7)

Q70 How often do you trim all the visible fat off the meat you eat (OR purchase pre-trimmed meat) and remove the skin from chicken before cooking?

- Always (1)
- Usually (2)
- Occasionally (3)
- Rarely (4)
- Never (5)
- I don't eat meat (6)

Q71 Which of the following cooking fats do you normally use?

- Butter (1)
- Margarine (8)
- Solid frying fat (2)
- Vegetable oil (3)
- Mono/Polyunsaturated oils (4)
- Sterol margarine (5)
- Spray oil (6)
- I don't use fat in cooking (7)

| Page Break |  |
| --- | --- |

Q72 Which of the following cooking methods do you commonly use when cooking? (You may select more than one.)

- Steaming (1)
- Poaching (2)
- Microwaving (3)
- Casseroles (4)
- Grilling (5)
- Stir Frying (6)
- Dry Roasting (7)
- Deep Frying (8)
- Shallow Frying (9)
- Roasting in Fat (10)

Q73 How often do you eat foods like pastries, cake, sweet biscuits or croissants?

- Six or more times per week (1)
- Three to five times per week (2)
- Once or twice per week (3)
- Less than once per week (4)
- Never, or hardly ever (5)

| Page Break |  |
| --- | --- |

Q74 How many days a week do you eat take-away style foods such as fried or BBQ chicken, fish and chips, Chinese, pizza, hamburgers etc?

- Five or more times per week (1)
- Three or four times per week (2)
- Once or twice per week (3)
- Less than once per week (4)
- Never, or hardly ever (5)

Q75 Which of the following foods do you eat most often as snacks between meals?

- Soy-based snacks, including soy yogurt, soy ice cream, soya biscuits (9)
- Chocolate bars (1)
- Crisps (chips)/fries (2)
- Roasted nuts (3)
- Sweet biscuits/cookies (4)
- Low fat (dairy) yoghurts (5)
- Olives, raw nuts, seeds (6)
- Fruit, dried fruit, vegetables (7)
- Other candy besides chocolate (8)
- Crackers (distinct from crisps and biscuits, including various savoury biscuits, rice crackers, etc.) (11)
- Other (10) ________________________________________________

| Page Break |  |
| --- | --- |

Q76 Do you currently follow a particular MS diet?

- Yes (1)
- No (2)

Display This Question:

If Q76 = Yes

Q77 Please select which diet you follow.

|  | Please indicate how rigorously you follow this diet | How long have you been following this diet? |
| --- | --- | --- |
|  |  |  |
| Overcoming Multiple Sclerosis (OMS) (1) | ▼ Not rigorously at all (1 ... Extremely rigorously (5) | ▼ Less than 3 months (1 ... 7 years or more (10) |
| Ashton Embry’s Best Bet Diet (2) | ▼ Not rigorously at all (1 ... Extremely rigorously (5) | ▼ Less than 3 months (1 ... 7 years or more (10) |
| Wahls’ Protocol Diet (3) | ▼ Not rigorously at all (1 ... Extremely rigorously (5) | ▼ Less than 3 months (1 ... 7 years or more (10) |
| McDougall Diet (4) | ▼ Not rigorously at all (1 ... Extremely rigorously (5) | ▼ Less than 3 months (1 ... 7 years or more (10) |
| Swank Diet (5) | ▼ Not rigorously at all (1 ... Extremely rigorously (5) | ▼ Less than 3 months (1 ... 7 years or more (10) |
| The Paleo diet (6) | ▼ Not rigorously at all (1 ... Extremely rigorously (5) | ▼ Less than 3 months (1 ... 7 years or more (10) |
| The 5:2 diet (7) | ▼ Not rigorously at all (1 ... Extremely rigorously (5) | ▼ Less than 3 months (1 ... 7 years or more (10) |
| Other (8) | ▼ Not rigorously at all (1 ... Extremely rigorously (5) | ▼ Less than 3 months (1 ... 7 years or more (10) |

| Page Break |  |
| --- | --- |

Q78 How often do you eat oily fish such as sardines, mackerel, herring, salmon, tuna or trout?

- Never (1)
- Less than once per week (2)
- Once or twice per week (3)
- Three or four times per week (4)
- Five or more times per week (5)

Q79 Do you take Omega-3 supplements?

- Yes (1)
- No (2)

Q80 Which type of Omega-3 supplements do you take?
(You may select more than one option)

- Marine-based oil (e.g. fish, krill, green-lipped mussel, algae, cod-liver oil) (1)
- Plant-based oil (e.g. flaxseed or linseed oil) (2)
- Other (3) ________________________________________________

Q81 In the last 12 months, what total dose of Omega-3 supplements (as standard strength marine-based or plant-based oil, measured in grams, mls or number of capsules) do you take on average per day?

________________________________________________________________

End of Block: Dietary Habits Questionnaire

Start of Block: Alcohol and smoking

Q82 Please refer to this guide for the definition of a standard drink:

 Full strength beer or premixed drinks with approx 5% alcohol: 285ml/9.6oz glass = 1
 Low alcohol beer with approx 2.5% alcohol: 285ml/9.6oz glass = 0.5
Wine with approx 13% alcohol: 100ml/3.4oz glass = 1
Spirits/liqueur with 35-40% alcohol: 30ml/1oz nip or equivalent mixed spirits = 1

Q83 Click to write the question text

|  | How often? | How much? |
| --- | --- | --- |
|  |  |  |
| How often do you usually drink alcohol, and how many standard drinks do you normally have on a day when you drink alcohol? (1) | ▼ Never (1 ... Every day of the week (10) | ▼ 0.5 (1 ... 10+ (11) |

| Page Break |  |
| --- | --- |

Q84 Do you smoke cigarettes or any tobacco products?

- Yes (1)
- No, but I used to smoke (2)
- No, I have never smoked (3)

Display This Question:

If Q84 = No, but I used to smoke

Q85 If you used to smoke, how long ago did you quit?

- Less than 6 months ago (1)
- 6 months to < 1 year ago (2)
- 1 year to < 2 years ago (3)
- 2 to < 3 years ago (4)
- 3 to < 4 years ago (5)
- 4 to < 5 years ago (6)
- 5 to < 10 years ago (7)
- > 10 years ago (8)

Display This Question:

If Q84 != No, I have never smoked

Q86 If you are a current or previous smoker, how many years have you smoked in your life all together?

- Less than 6 months (4)
- 6 months to < 1 year (5)
- 1 year to < 2 years (6)
- 2 to < 3 years (7)
- 3 to < 4 years (8)
- 4 to < 5 years (9)
- 5 to < 10 years (10)
- > 10 years (11)

Display This Question:

If Q84 != No, I have never smoked

Q87 If you are a current or previous smoker, on average, how many cigarettes do/did you smoke each day?

- Less than 1 per day (1)
- 1 - 5 per day (2)
- 6 - 10 per day (3)
- 11 - 15 per day (4)
- 16 - 20 per day (5)
- More than 20 per day (6)

End of Block: Alcohol and smoking

Start of Block: Medication

Q203
The following two medications are **first generation disease modifying drugs** (with trade names in brackets) used to manage MS. Please select the medication you currently take, or have previously taken and how long you have used this medication in total. Please skip over any medications you have never used.

|  | Select currently taking or previously taken | | Length of time taken |
| --- | --- | --- | --- |
|  | I am currently taking this (1) | I have previously taken this (2) |  |
| Interferons (Avonex®, Betaferon®, Extavia®, Rebif) (14) |  |  | ▼ Less than ( |
| Glatiramer Acetate (Copaxone®) (29) |  |  | ▼ Less than ( |

Q88
The following is an alphabetic list of **second generation disease modifying drugs** (with trade names in brackets) used to manage MS. Please select the medications you currently take, or have previously taken and how long you have used this medication in total. Please skip over any medications you have never used.

|  | Select currently taking or previously taken | | Length of time taken |
| --- | --- | --- | --- |
|  | I am currently taking this (1) | I have previously taken this (2) |  |
| Alemtuzumab (Campath®, Lemtrada®) (2) |  |  | ▼ Less than ( |
| BG-12 (Dimethyl Fumarate, Tecfidera®) (5) |  |  | ▼ Less than ( |
| Cladribine (Leustat, Movectro, Movectra) (6) |  |  | ▼ Less than ( |
| Daclizumab (Zenapax®) (8) |  |  | ▼ Less than ( |
| Fingolimod (FTY-720, Gilenya®) (11) |  |  | ▼ Less than ( |
| Laquinimod (Nerventra®) (15) |  |  | ▼ Less than ( |
| Natalizumab (Tysabri®) (21) |  |  | ▼ Less than ( |
| Ocrelizumab (Ocrevus) (22) |  |  | ▼ Less than ( |
| Peginterferon Beta-1a (Plegridy®) (23) |  |  | ▼ Less than ( |
| Rituximab (Rituxan®, Ocrelizumab (25) |  |  | ▼ Less than ( |
| Teriflunomide (Aubagio®) (28) |  |  | ▼ Less than ( |

Q204
The following two medications are **steroid medications** (with trade names in brackets) used to manage MS. Please select the medication you currently take, or have previously taken and how long you have used this medication in total. Please skip over any medications you have never used.

|  | Select currently taking or previously taken | | Length of time taken |
| --- | --- | --- | --- |
|  | I am currently taking this (1) | I have previously taken this (2) |  |
| Adrenocorticotropic hormone (Acthar®) (14) |  |  | ▼ Less than ( |
| Prednisone, Prednisolone (29) |  |  | ▼ Less than ( |

Q206
The following medications are **general immunosuppresents** (some with trade names in brackets) used to manage MS. Please select the medication you currently take, or have previously taken and how long you have used this medication in total. Please skip over any medications you have never used.

|  | Select currently taking or previously taken | | Length of time taken |
| --- | --- | --- | --- |
|  | I am currently taking this (1) | I have previously taken this (2) |  |
| Azathioprine (Imuran®, Azasan®) (14) |  |  | ▼ Less than ( |
| Cyclophosphamide (Cytoxan®, Revimmune) (29) |  |  | ▼ Less than ( |
| Methotrexate (Folex, Matrex®, Rheumatrex®, Trexall®) (30) |  |  | ▼ Less than ( |
| Mitoxantrone (Novantrone) (31) |  |  | ▼ Less than ( |
| Mycophenalate (32) |  |  | ▼ Less than ( |

Q207
The following medications are **other medications/procedures** (some with trade names in brackets) used to manage MS. Please select any you currently take/use, or have previously taken/used and how long you have used these in total. Please skip over any you have never used.

|  | Select currently taking or previously taken | | Length of time taken |
| --- | --- | --- | --- |
|  | I am currently taking this (1) | I have previously taken this (2) |  |
| Immunoglobulins IVIG (Gamma globulin) (14) |  |  | ▼ Less than ( |
| Plasmapheresis / Plasma exchange (29) |  |  | ▼ Less than ( |
| Low-dose Naltrexone (LDN) (30) |  |  | ▼ Less than ( |
| Minocycline (Minomycin) (31) |  |  | ▼ Less than ( |
| Baclofen (Kemnstro, Lioresal) (32) |  |  | ▼ Less than ( |
| Fampridine (Ampyra) (33) |  |  | ▼ Less than ( |
| Statins (Crestor®, Lescol,® Lipitor®, Pravachol®, Zocor®) (34) |  |  | ▼ Less than ( |
| Fibrates (Clofibrate, Atromid-S®, Fenofibrate, Lipidil®, Gemfibrozil, Bezafibrate, Bezalip®) (35) |  |  | ▼ Less than ( |
| Other: (36) |  |  | ▼ Less than ( |

| Page Break |  |
| --- | --- |

Q89 Please indicate if you regularly take prescription medication, over-the-counter (non-prescription) medication or herbal remedies for the following conditions associated with MS:

|  |  |  |  |
| --- | --- | --- | --- |
|  | Prescription (1) | Over-the-counter (1) | Herbal Remedy (1) |
| Depression (1) |  |  |  |
| Anxiety (2) |  |  |  |
| Headaches (3) |  |  |  |
| Pain (other than headaches) (4) |  |  |  |
| Fatigue (5) |  |  |  |
| Difficulty sleeping at night (6) |  |  |  |
| Bladder problems (7) |  |  |  |
| Bowel problems (8) |  |  |  |
| Spasticity (9) |  |  |  |
| Other (10) |  |  |  |

End of Block: Medication

Start of Block: Physical Activity

| 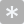 | 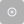 | 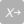 | 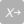 |
| --- | --- | --- | --- |

Q148
During a typical 7-Day period (a week), how many times on the average do you do the following kinds of exercise for more than 15 minutes during your free time (write on each line the appropriate number).

|  | How many times per week? (1) |
| --- | --- |
| MILD/LIGHT EXERCISE (MINIMAL EFFORT) (e.g., yoga, archery, fishing from river bank, bowling, horseshoes, golf, snow-mobiling, easy walking) (Q148_3) |  |
| MODERATE EXERCISE (NOT EXHAUSTING) (e.g., fast walking, baseball, tennis, easy bicycling, volleyball, badminton, easy swimming, alpine skiing, popular and folk dancing)   (Q148_2) |  |
| STRENUOUS EXERCISE (HEART BEATS RAPIDLY) (e.g., running, jogging, hockey, football, soccer, squash, basketball, cross country skiing, judo, roller skating, vigorous swimming, vigorous long distance bicycling)   (Q148_1) |  |

End of Block: Physical Activity

Start of Block: Sun Exposure

Q94 Sun exposure

|  | How many days per week were you out in the sun? | How long on average were you out in the sun on such a day? |
| --- | --- | --- |
|  |  |  |
| During last summer (1) | ▼ 0 days (1 ... Every day (8) | ▼ Not at all (1 ... > 6 hours (8) |
| During last winter (2) | ▼ 0 days (1 ... Every day (8) | ▼ Not at all (1 ... > 6 hours (8) |

Q95

|  | In summer | In winter |
| --- | --- | --- |
|  |  |  |
| Sunscreen on the majority of your exposed skin (1) | ▼ Never (1 ... Always (4) | ▼ Never (1 ... Always (4) |
| Clothes that covered most of your body (e.g. long sleeve shirt and long pants) (2) | ▼ Never (1 ... Always (4) | ▼ Never (1 ... Always (4) |
| Clothes that exposed much of your body (e.g. shorts and t-shirt, or swimwear) (3) | ▼ Never (1 ... Always (4) | ▼ Never (1 ... Always (4) |

| Page Break |  |
| --- | --- |

Q96 In the last 12 months, about how often have you got adequate sun exposure (10-15 minutes of sunlight on a day with UV index of 7 {more or less if the UV index is lower or higher})?

▼ Never (1) ... Unsure (7)

Q97 Do you intentionally get sun exposure to raise your vitamin D level?

- Yes (1)
- No (2)

Q98 What is the colour of your untanned skin?

▼ Very light (1) ... Very dark (6)

| Page Break |  |
| --- | --- |

Q100 Do you take a vitamin D supplement?

- Yes (1)
- No (2)

Q101 Please specify the dose of vitamin D, how often and for how many months of the year you take it. If you take the same dose at the same frequency all year, please only complete row 1. If your dosage or frequency is different throughout the year, for example, if you supplement with a different amount in summer to winter, please record your supplement information for summer in row 1 and for winter in row 2, etc.

|  | Dose of Vitamin D (international units) | How often do you take this dose on average? | How many months of the year do you take this dose? |
| --- | --- | --- | --- |
|  |  |  |  |
| Regular Vitamin D supplementation (1) | ▼ Unsure (1 ... 500,000 or more (19) | ▼ Single time (1 ... Daily (10) | ▼ 1 (1 ... 12 (12) |
| Alternative Vitamin D supplementation (2) | ▼ Unsure (1 ... 500,000 or more (19) | ▼ Single time (1 ... Daily (10) | ▼ 1 (1 ... 12 (12) |

Q102 How long have you been taking vitamin D supplements?

- Less than ( (1)
- 6 months to < 12 months (2)
- 1 year to < 2 years (3)
- 2 to < 3 years (4)
- 3 to < 4 years (5)
- 4 to < 5 years (6)
- 5 to < 10 years (7)
- More than 10 years (8)

End of Block: Sun Exposure

Start of Block: Stress Management

Q103 On average in the last 12 months, how often have you meditated?

- Never (1)
- Less than once per week (2)
- 1 day a week (3)
- 2 days per week (4)
- 3 days per week (5)
- 4 days per week (6)
- 5 days per week (7)
- 6 days per week (8)
- Every day (9)

Q104 On average, for how long do you meditate (in minutes) each time?

- less than 10 minutes (1)
- 10 (2)
- 15 (3)
- 20 (4)
- 30 (5)
- 40 (6)
- 50 (7)
- 60 (8)
- More than 60 minutes (9)

Q105 Apart from meditation, do you regularly participate in any activities that help manage stress and promote relaxation. Choose from below.

- Yoga (1)
- Qigong (2)
- Art (3)
- Music Therapy (4)
- Prayer (6)
- Other (5) ________________________________________________

End of Block: Stress Management

Start of Block: Vision

Q109 Please read all of the categories, and check the single category which best describes your overall visual condition (with glasses if you use them) over the past month. Compare your current condition to your vision before you developed MS.

- 0. Normal Vision: Functionally normal; no limitations on activity or lifestyle. (MS has not affected my vision/ I wear glasses but otherwise my vision is normal.) (1)
- 1. Minimal Visual Disability (My vision is normal, but it has been affected in the past by MS.) (2)
- 2. Mild Visual Disability (I have visual symptoms of either blurred or double vision, but I am still able to do all of my usual activities.) (3)
- 3. Moderate Visual Disability (Because of my visual problems, I have been forced to give up some of my usual activities, but after a period of rest I can usually return to these activities.) (4)
- 4. Severe Visual Disability (I am no longer able to maintain my original lifestyle because of my vision and rest does not seem to help my vision, I can no longer drive a car because of my vision.) (5)
- 5. Total Visual Disability (I am essentially blind. I cannot read, even with aids.) (6)

| Page Break |  |
| --- | --- |

End of Block: Vision

Start of Block: Work Productivity and Activity Impairment Questionnaire

Q158 The following ask about the effect of your multiple sclerosis on your ability to work and perform normal activities. Please fill in in the blanks of move the slider, as indicated.

Q157 In relation to your work status, please choose from the following categories:

- Working for Pay (1)
- Working in the home by choice (2)
- Volunteering (3)
- Studying (4)
- Temporarily not working but looking for work (5)
- Not working because of age (6)
- Not working because of disease (7)
- Never worked before (8)
- Other (9) ________________________________________________

Q159 The next set of questions **refer to the past 7 days, not including today.**

Q162 During the past seven days, how many hours did you miss from work because of problems associated with your multiple sclerosis? Include hours you missed on sick days, times you went in late, left early, etc., because of your multiple sclerosis. Do not include time you missed to participate in this study.

▼ 0 to < 1 hour (4) ... over 24 hours (8)

Q161 During the past seven days, how many hours did you miss from work for any other reason, such as annual leave, holidays, time off to participate in this study?

▼ 0 - < 1 hour (4) ... over 24 hours (8)

Q163 During the past seven days, how many hours did you actually work?

▼ 0 - < 1 hour (7) ... over 24 hours (8)

Q164 During the past seven days, how much did your multiple sclerosis affect your productivity while you were working?

 *Think about days you were limited in the amount or the kind of work could do, days you accomplished less than you would like, or days you could not do your work as carefully as usual. If your multiple sclerosis affected your work only a little, choose a low number. Choose a high number if multiple sclerosis affected your work a great deal.*

|  | Multiple sclerosis had no affect on my work | Multiple sclerosis completely prevented me from working |
| --- | --- | --- |

|  | 0 | 1 | 2 | 3 | 4 | 5 | 6 | 7 | 8 | 9 | 10 |
| --- | --- | --- | --- | --- | --- | --- | --- | --- | --- | --- | --- |

| 1 () | 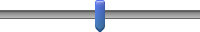 |
| --- | --- |

Q166 During the past 7 days, how much did your multiple sclerosis affect your ability to perform your normal daily activities, other than work at a job?

 *By normal activities, we mean the usual activities you perform, such as working around the house, shopping, childcare, exercise, studying, etc. Think about times you were limited in the amount or kind of activities you could perform and times you accomplished less than you would like. Choose a lower number if multiple sclerosis affected your activities only a little, or choose high if multiple sclerosis had a great impact on your activities.*

|  | Multiple sclerosis had no affect on my daily activities | Multiple sclerosis completely prevented me from daily activities |
| --- | --- | --- |

|  | 0 | 1 | 2 | 3 | 4 | 5 | 6 | 7 | 8 | 9 | 10 |
| --- | --- | --- | --- | --- | --- | --- | --- | --- | --- | --- | --- |

| 1 () | 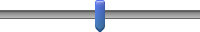 |
| --- | --- |

End of Block: Work Productivity and Activity Impairment Questionnaire

Start of Block: Assessment of Quality of Life - 8 dimension

Q167 How much energy do you have to do the things you want to do? I am:

- Always full of energy (1)
- Usually full of energy (2)
- Occasionally energetic (3)
- Usually tired and lacking energy (4)
- Always tired and lacking energy (5)

Q168 How often do you feel socially excluded or left out?

- Never (1)
- Rarely (2)
- Sometimes (3)
- Often (4)
- Always (5)

Q169 How easy or difficult is it for you to get around by yourself outside your place of residence (eg. to go shopping, visiting)?

- Getting around is enjoyable and easy (1)
- I have no difficulty getting around outside my place of residence (2)
- A little difficulty (3)
- Moderate difficulty (4)
- A lot of difficulty (5)
- I cannot get around unless someone is there to help me (6)

Q170 Does your health affect your role in your community (residential, sporting, religious or cultural activities)?

- My role in the community is unaffected by my health (1)
- There are some parts of my community role I cannot carry out (2)
- There are many parts of my community role I cannot carry out (3)
- I cannot carry out any part of my community role (4)

Q171 How often do you feel sad?

- Never (1)
- Rarely (2)
- Sometimes (3)
- Usually (4)
- Nearly all of the time (7)

Q173 How often do you feel sad?

- Never (1)
- Rarely (2)
- Sometimes (3)
- Usually (4)
- Nearly all of the time (7)

Q172 How much confidence do you have in yourself?

- Complete confidence (1)
- A lot (2)
- A moderate amount (3)
- A little (4)
- None at all (7)

Q174 Do you normally feel calm and tranquil, or agitated?

- I am always calm and tranquil (1)
- I am usually calm and tranquil (2)
- I am sometimes calm and tranquil, sometimes agitated (3)
- I am usually agitated (4)
- I am always agitated (7)

Q175 Does your health affect your relationship with your family?

- My role in the family is unaffected by my health (1)
- There are some part of my family role I cannot carry out (2)
- There are many parts of my family role I cannot carry out (3)
- I cannot carry out any part of my family role (4)

Q176 How satisfying are your close relationships (family and friends)?

- Very satisfying (1)
- Satisfying (2)
- Neither satisfying nor dissatisfying (3)
- Dissatisfying (4)
- Unpleasant (8)
- Very unpleasant (9)

Q177 How well do you communicate with others (talking, texting, signing, being understood by other and understanding them)?

- I have no trouble being understood (1)
- I have some difficulty being understood by people who do not know me (2)
- I am understood only by people who know me (3)
- I cannot adequately communicate with others (4)

Q178 How often do you have trouble sleeping?

- Never (1)
- Almost never (2)
- Sometimes (3)
- Often (4)
- All the time (10)

Q179 How often do you feel worthless?

- Never (1)
- Almost never (2)
- Sometimes (3)
- Usually (4)
- All the time (10)

Q180 How often do you feel angry?

- Never (1)
- Almost never (2)
- Sometimes (3)
- Usually (4)
- All the time (10)

Q181 How easy or difficult is it for you to move around (using any aids or equipment you need eg. a wheelchair, frame or stick)?

- I am very mobile (1)
- I have no difficulty with mobility (2)
- I have some difficulty with mobility (for example, going uphill) (3)
- I have difficulty with mobility. I can go short distances only (4)
- I have a lot of difficulty with mobility. I need someone to help me (10)
- I am bedridden (11)

Q182 Do you ever feel like hurting yourself?

- Never (1)
- Rarely (2)
- Sometimes (3)
- Often (4)
- All the time (10)

Q183 How enthusiastic do you feel?

- Extremely (1)
- Very (2)
- Somewhat (3)
- Not much (4)
- Not at all (10)

Q184 How often did you feel worried in the last 7 days?

- Never (1)
- Occasionally (2)
- Sometimes (3)
- Often (4)
- All the time (10)

Q185 How difficult is it for you to wash, toilet, dress yourself, eat or care for your appearance?

- These things are very easy for me to do (1)
- I have no real difficulty in doing these things (2)
- I find some of these things difficult, but I manage to do them on my own (3)
- Many of these things are difficult, and I need help to do them (4)
- I cannot do these things at all by myself (10)

Q186 How often do you feel happy?

- All the time (1)
- Most of the time (2)
- Some of the time (3)
- Almost never (4)
- Never (10)

Q187 How much do you feel you can cope with life's problems?

- All the time (1)
- Most of the time (2)
- Some of the time (3)
- Almost never (4)
- Never (10)

Q188 How much pain or discomfort do you experience?

- None at all (1)
- I have moderate pain (2)
- I suffer from severe pain (3)
- I suffer unbearable pain (4)

Q189 How often do you enjoy your close relationships (family and friends)?

- Immensely (1)
- A lot (2)
- A little (3)
- Not much (4)
- Not at all (12)

Q190 How often does pain interfere with your usual activities?

- Never (1)
- Rarely (2)
- Sometimes (3)
- Often (4)
- All the time (12)

Q191 How often do you feel pleasure?

- Always (1)
- Usually (2)
- Sometimes (3)
- Rarely (4)
- Never (12)

Q192 How much of a burden do you feel towards other people?

- Not at all (1)
- A little (2)
- A moderate amount (3)
- A lot (4)
- Totally (12)

Q193 How content are you with your life?

- Extremely (1)
- Mainly (2)
- Moderately (3)
- Slightly (4)
- Not at all (12)

Q194 How well can you see (using your glasses or contact lenses if they are needed)?

- I have excellent sight (1)
- I see normally (2)
- I have some difficulty seeing things sharply (eg. small print, objects in the distance, or television). (3)
- I have a lot of difficulty seeing sharply (4)
- I only see general shapes (12)
- I am completely blind (13)

Q195 How often do you feel in control of your life?

- Always (1)
- Often (2)
- Some of the time (3)
- Rarely (4)
- Never (12)

Q196 How much help do you need with jobs around your place of residence (eg. preparing food, cleaning, gardening)?

- I can do all these tasks very easily without any help (1)
- I can do these tasks relatively easily without help (2)
- I can do these tasks only very slowly without help (3)
- I cannot do most of these tasks unless I have help (4)
- I can do none of these tasks by myself (12)

Q197 How often do you feel socially isolated?

- Never (1)
- Rarely (2)
- Some of the time (3)
- Often (4)
- Always (12)

Q198 How well can you hear (using your hearing aid if needed)?

- I have excellent hearing (1)
- I hear normally (2)
- I have some difficulty hearing or I do not hear clearly (eg. when there is background noise) (3)
- I have difficulty hearing things clearly. Often I do not understand what is said. Usually I do not take part in conversations because I cannot hear what is being said (4)
- I hear very little (12)
- I am completely deaf (14)

Q199 How often do you feel depressed?

- Never (1)
- Almost never (2)
- Sometimes (3)
- Often (4)
- Very Often (12)
- All the time (16)

Q200 How happy are you with your close and intimate relationships?

- Very happy (1)
- Generally happy (2)
- Neither happy nor unhappy (3)
- Generally unhappy (4)
- Very unhappy (12)

Q201 How often did you feel in despair in the last 7 days?

- Never (1)
- Occasionally (2)
- Some of the time (3)
- Often (4)
- All of the time (12)

End of Block: Assessment of Quality of Life - 8 dimension

Start of Block: Multiple Sclerosis Quality of Life MSQOL -54

Q220 In general, would you say your health is:

- Excellent (1)
- Very Good (2)
- Good (3)
- Fair (4)
- Poor (5)

Q221
Compared to one year ago, how would you rate your health in general now?

- Much better now than one year ago. (1)
- Somewhat better now than one year ago. (2)
- About the same. (3)
- Somewhat worse now than a one year ago. (4)
- Much worse now than one year ago. (5)

| Page Break |  |
| --- | --- |

Q222
***The following questions are about activities you might do during a typical***
***day. Does your health limit you in these activities? If so, how much?***

Q223 Vigorous activities, such as running, lifting heavy objects, participating in strenuous sports.

- Yes, limited a lot. (1)
- Yes, limited a little. (2)
- No, not limited at all. (3)

Q224 Moderate activities, such as moving a table, pushing a vacuum cleaner, bowling, or playing golf.

- Yes, limited a lot. (1)
- Yes, limited a little. (2)
- No, not limited at all. (3)

Q225 Lifting or carrying groceries.

- Yes, limited a lot. (1)
- Yes, limited a little. (2)
- No, not limited at all. (3)

Q226 Climbing several flights of stairs.

- Yes, limited a lot. (1)
- Yes, limited a little. (2)
- No, not limited at all. (3)

Q227 Climb one flight of stairs.

- Yes, limited a lot. (1)
- Yes, limited a little. (2)
- No, not limited at all. (3)

Q228 Bending, kneeling or stooping.

- Yes, limited a lot. (1)
- Yes, limited a little. (2)
- No, not limited at all. (3)

Q229 Walking more than a mile.

- Yes, limited a lot. (1)
- Yes, limited a little. (2)
- No, not limited at all. (3)

Q230 Walking several blocks.

- Yes, limited a lot. (1)
- Yes, limited a little. (2)
- No, not limited at all. (3)

Q231 Walking one block.

- Yes, limited a lot. (1)
- Yes, limited a little. (2)
- No, not limited at all. (3)

Q232 Bathing and dressing yourself.

- Yes, limited a lot. (1)
- Yes, limited a little. (2)
- No, not limited at all. (3)

| Page Break |  |
| --- | --- |

Q233 During the past 4 weeks, have you had any of the following problems with your work or other regular daily activities as a result of your physical health?

Q234 Cut down on the time you could spend on work or other activities

- Yes (28)
- No (29)

Q283 Accomplished less than you would like

- Yes (28)
- No (29)

Q236 Were limited in the kind of work or other activities

- Yes (28)
- No (29)

Q280 Had difficulty performing the work or other activities (for example, it took extra effort)

- Yes (28)
- No (29)

Q282 ***During the past 4 weeks, have you had any of the following problems with your work or other regular daily activities as a result of any emotional problems (such as feeling depressed or anxious)?***

Q281 Cut down on the amount of time you could spend on work or other activities

- Yes (28)
- No (29)

Q235 Accomplished less than you would like

- Yes (28)
- No (29)

Q284 Didn't do work or other activities as carefully as usual

- Yes (28)
- No (29)

| Page Break |  |
| --- | --- |

Q237 During the past 4 weeks, to what extent has your physical health or emotional problems interfered with your normal social activities with family, friends, neighbors, or groups?

- Not at all (1)
- Slightly (2)
- Moderately (3)
- Quite a bit (4)
- Extremely (5)

Q238 How much bodily pain have you had during the past 4 weeks?

- None (1)
- Very mild (2)
- Mild (3)
- Moderate (4)
- Severe (5)
- Very severe (6)

Q239 During the past 4 weeks, how much did pain interfere with your normal work (including both work outside the home and housework)?

- Not at all (1)
- A little bit (2)
- Moderately (3)
- Quite a bit (4)
- Extremely (5)

| Page Break |  |
| --- | --- |

Q240
***These questions are about how you feel and how things have been with you***
***during the past 4 weeks. For each question, please give the one answer that***

***comes closest to the way you have been feeling.***
***How much of the time during the past 4 weeks...***

Q241 Do you feel full of pep?

▼ All of the time (1) ... None of the time (6)

Q242 Have you been a very nervous person?

▼ All of the time (1) ... None of the time (6)

Q243 Have you been so down that nothing can cheer you up?

▼ All of the time (1) ... None of the time (6)

Q244 Have you felt calm and peaceful?

▼ All of the time (1) ... None of the time (6)

Q245 Did you have a lot of energy?

▼ All of the time (1) ... None of the time (6)

Q246 Have you felt downhearted and blue?

▼ All of the time (1) ... None of the time (6)

Q247 Did you feel warn out?

▼ All of the time (1) ... None of the time (6)

Q248 Have you been a happy person?

▼ All of the time (1) ... None of the time (6)

Q249 Did you feel tired?

▼ All of the time (1) ... None of the time (6)

Q250 Did you feel rested on waking in the morning?

▼ All of the time (1) ... None of the time (6)

| Page Break |  |
| --- | --- |

Q251 During the past 4 weeks, how much of the time has your physical health or emotional problems interfered with your social activities (like visiting with friends, relatives, etc.)?

- All of the time (1)
- Most of the time (2)
- Some of the time (3)
- A little of the time (4)
- None of the time (5)

Q252
***How TRUE or FALSE is each of the following statements for you.***

Q253 I seem to get sick a little easier than other people

▼ Definitely true (1) ... Definitely false (5)

Q254 I am as healthy as anybody I know

▼ Definitely true (1) ... Definitely false (5)

Q255 I expect my health to get worse

▼ Definitely true (1) ... Definitely false (5)

Q256 My health is excellent

▼ Definitely true (1) ... Definitely false (5)

| Page Break |  |
| --- | --- |

Q257
***How much of the time during the past 4 weeks...***

Q258 Were you discouraged by your health problems?

▼ All of the time (1) ... None of the time (6)

Q259 Were you frustrated about your health?

▼ All of the time (1) ... None of the time (6)

Q260 Was your health a worry in your life?

▼ All of the time (1) ... None of the time (6)

Q261 Did you feel weighed down by your health problems?

▼ All of the time (1) ... None of the time (6)

Q262 ***How much of the time during the past 4 weeks...***

Q263 Have you had difficulty concentrating and thinking?

▼ All of the time (1) ... None of the time (6)

Q264 Did you have trouble keeping your attention on an activity for long?

▼ All of the time (1) ... None of the time (6)

Q265 Have you had trouble with your memory?

▼ All of the time (1) ... None of the time (6)

Q266 Have others, such as family members or friends, noticed that you have troubles with your memory or problems with your concentration?

▼ All of the time (1) ... None of the time (6)

| Page Break |  |
| --- | --- |

Display This Question:

If Q17 != Slightly

Q267 ***The next set of questions are about your sexual function and your satisfaction with your sexual function. Please answer as accurately as possible about your function during the last 4 weeks only.***

 ***How much of a problem was each of the following for you during the past 4 weeks?***

Display This Question:

If Q17 != Slightly

Q268 Lack of sexual interest

▼ Not a problem (1) ... Very much a problem (4)

Display This Question:

If Q17 != Slightly

Q269 Difficulty getting or keeping an erection

▼ Not a problem (1) ... Very much a problem (4)

Display This Question:

If Q17 != Slightly

Q270 Difficulty having an orgasm

▼ Not a problem (1) ... Very much a problem (4)

Display This Question:

If Q17 != Slightly

Q271 Ability to satisfy sexual partner

▼ Not a problem (1) ... Very much a problem (4)

Display This Question:

If Q17 != Not at all

Q272
***The next set of questions are about your sexual function and your satisfaction with your sexual function. Please answer as accurately as possible about your function during the last 4 weeks only.***

 How much of a problem was each of the following for you during the past 4 weeks?

Display This Question:

If Q17 != Not at all

Q273 Lack of sexual interest

▼ Not a problem (1) ... Very much a problem (4)

Display This Question:

If Q17 != Not at all

Q274 Inadequate lubrication

▼ Not a problem (1) ... Very much a problem (4)

Display This Question:

If Q17 != Not at all

Q275 Difficulty having an orgasm

▼ Not a problem (1) ... Very much a problem (4)

Display This Question:

If Q17 != Not at all

Q276 Ability to satisfy sexual partner

▼ Not a problem (1) ... Very much a problem (4)

Q277 Overall, how satisfied were you with your sexual function during the past 4 weeks?

- Very satisfied (1)
- Somewhat satisfied (2)
- Neither satisfied nor dissatisfied (3)
- Somewhat dissatisfied (4)
- Very dissatisfied (5)

| Page Break |  |
| --- | --- |

Q278 During the past 4 weeks, to what extent do you have problems with your bowel or bladder function, which interfered with your normal social activities with family, friends, neighbors, or groups?

- Not at all (1)
- Slightly (2)
- Moderately (3)
- Quite a bit (4)
- Extremely (5)

Q279 During the past 4 weeks, how much did pain interfere with your enjoyment of life?

- Not at all (1)
- Slightly (2)
- Moderately (3)
- Quite a bit (4)
- Extremely (5)

| Page Break |  |
| --- | --- |

| 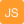 |
| --- |

Q280 Overall, how would you rate your own quality-of-life?

|  | Worst Possible | Best Possible |
| --- | --- | --- |

|  | 0 | 1 | 2 | 3 | 4 | 5 | 6 | 7 | 8 | 9 | 10 |
| --- | --- | --- | --- | --- | --- | --- | --- | --- | --- | --- | --- |

| 1 () | 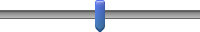 |
| --- | --- |

Q281 Which best describes how you feel about your life as a whole?

- Terrible (1)
- Unhappy (2)
- Mostly dissatisfied (3)
- Mixed - about equally satisfied and dissatisfied (4)
- Mostly satisfied (5)
- Pleased (6)
- Delighted (7)

End of Block: Multiple Sclerosis Quality of Life MSQOL -54
