# Supplementary material for: The Feasibility of a Web-Based Educational Lifestyle Program for People With Multiple Sclerosis: A Randomized Controlled Trial
Source: Front Public Health. 2022 Apr 27;10:852214. doi: 10.3389/fpubh.2022.852214 (PMC9092338; doi:10.3389/fpubh.2022.852214)
Supplement: Supplementary Figure 3 — Time series plot of users accessing the courses in both intervention and standard-care. [file Table_2.DOCX]

Follow-up Survey

Start of Block: Default Question Block

Q1 This information will be used to link your responses across different time points (please note that this information is confidential and you will not be identified in any results or publications and your information will not be given to any third parties). All fields are required.

- First name (1) ________________________________________________
- Surname (2) ________________________________________________
- Primary email address (3) ________________________________________________
- Landline phone (include country code) (4) ________________________________________________
- Mobile phone (5) ________________________________________________

End of Block: Default Question Block

Start of Block: Screening

Q26 Did you commence the online course (completed at least 1 module)?

- Yes (1)
- No (2)

End of Block: Screening

Start of Block: Why

Display This Question:

If Q26 = No

Q27 What was the reason you did not commence the course?

- Did not have the time (1)
- Lost interest since signing up (2)
- Illness (3)
- I logged in and did not like the look of the course (4)
- Other (5) ________________________________________________

Skip To: End of Survey If Q27 , Did not have the time Is Displayed

End of Block: Why

Start of Block: Learnability

Q4 It was easy to learn how to use this course

- Strongly agree (1)
- Agree (2)
- Neither agree nor disagree (3)
- Disagree (4)
- Strongly disagree (5)

Q6 I needed assistance during this course

- Strongly agree (1)
- Agree (2)
- Neither agree nor disagree (3)
- Disagree (4)
- Strongly disagree (5)

Q7 I had to learn new skills to use this course

- Strongly agree (1)
- Agree (2)
- Neither agree nor disagree (3)
- Disagree (4)
- Strongly disagree (5)

Q8 I felt confident using this course

- Strongly agree (1)
- Agree (2)
- Neither agree nor disagree (3)
- Disagree (4)
- Strongly disagree (5)

Q11 Please expand upon your thoughts:

________________________________________________________________

________________________________________________________________

________________________________________________________________

________________________________________________________________

________________________________________________________________

End of Block: Learnability

Start of Block: Accessibility

Q18 It was easy to login to this course

- Strongly agree (1)
- Agree (2)
- Neither agree nor disagree (3)
- Disagree (4)
- Strongly disagree (5)

Q19 I found moving through the modules easy

- Strongly agree (1)
- Agree (2)
- Neither agree nor disagree (3)
- Disagree (4)
- Strongly disagree (5)

Q22 I found it difficult to find the right place to click the mouse

- Always (1)
- Often (2)
- Sometimes (3)
- Rarely (4)
- Never (5)

Q20 The colour scheme was good

- Strongly agree (1)
- Agree (2)
- Neither agree nor disagree (3)
- Disagree (4)
- Strongly disagree (5)

Q21 The font size was appropriate

- Strongly agree (1)
- Agree (2)
- Neither agree nor disagree (3)
- Disagree (4)
- Strongly disagree (5)

Q23 Please expand upon your thoughts:

________________________________________________________________

________________________________________________________________

________________________________________________________________

________________________________________________________________

________________________________________________________________

End of Block: Accessibility

Start of Block: Desirability

Q10 The course should have been:

- Longer than 6 weeks (1)
- It was the right length (2)
- Shorter than 6 weeks (3)

Q9 Modules should have been released:

- More frequently than twice per week (1)
- It was the right timing (2)
- Less frequently than twice per week (3)

Q11 I liked the written content

- Strongly agree (1)
- Agree (2)
- Neither agree nor disagree (3)
- Disagree (4)
- Strongly disagree (5)

Q12 I liked the video content

- Strongly agree (1)
- Agree (2)
- Neither agree nor disagree (3)
- Disagree (4)
- Strongly disagree (5)

Q13 I liked the interactive content

- Strongly agree (1)
- Agree (2)
- Neither agree nor disagree (3)
- Disagree (4)
- Strongly disagree (5)

| Page Break |  |
| --- | --- |

Q14 I found the forum useful

- Strongly agree (1)
- Agree (2)
- Neither agree nor disagree (3)
- Disagree (4)
- Strongly disagree (5)

Q15 I felt part of a larger group during the course

- Strongly agree (1)
- Agree (2)
- Neither agree nor disagree (3)
- Disagree (4)
- Strongly disagree (5)

Q24 Technical issues made the course difficult

- Strongly agree (1)
- Agree (2)
- Neither agree nor disagree (3)
- Disagree (4)
- Strongly disagree (5)

Q16 Please expand upon your thoughts:

________________________________________________________________

________________________________________________________________

________________________________________________________________

________________________________________________________________

________________________________________________________________

End of Block: Desirability

Start of Block: Block 4

Q25 Please select the following areas you have made changes to as a result of the course:

- Diet (1)
- Physical activity (2)
- Stress/mindfulness (3)
- Sun exposure/vitamin D (4)
- Family prevention (5)
- I haven't made any changes to mine or other's lifestyle (6)
- I have made lifestyle changes but not due to the course (7)

End of Block: Block 4
