# Supplementary material for: The Feasibility of a Web-Based Educational Lifestyle Program for People With Multiple Sclerosis: A Randomized Controlled Trial
Source: Front Public Health. 2022 Apr 27;10:852214. doi: 10.3389/fpubh.2022.852214 (PMC9092338; doi:10.3389/fpubh.2022.852214)
Supplement: Supplementary Table 1 — Devices used to access the courses. [file Data_Sheet_1.docx]

**Supplementary Table 1. Devices used to access the courses.**

| Device used to access course, n | **Intervention (n=15)** | **Standard-care (n=16)** |
| --- | --- | --- |
| Desktop | 21 | 20 |
| Smartphone | 5 | 2 |
| Tablet | 1 | 2 |

**Supplementary Table 2. Respondent answers to follow-up survey on length of course and frequency of module release.**

|  | Longer than 6 weeks | It was the right length | Shorter than 6 weeks |
| --- | --- | --- | --- |
| The course should have been: | 5 (28%) | 13 (72%) | 0 (0%) |
|  | More frequently | It was the right frequency | Less frequently |
| Modules should have been released: | 2 (11%) | 15 (83%) | 1 (6%) |
